# Supplementary material for: Data quality in centenarian research: The proxy-centenarian relationship and item nonresponse in the SWISS100 study
Source: PLoS One. 2025 Jan 27;20(1):e0311847. doi: 10.1371/journal.pone.0311847 (PMC11771874; doi:10.1371/journal.pone.0311847)
Supplement: S1 Table — (PDF) [file pone.0311847.s001.pdf]

S1 Tab. Variance inflation factors and tolerance criteria

	M1		M2		M3		M4		M5	
	VIF	TC	VIF	TC	VIF	TC	VIF	TC	VIF	TC
Proxy-centenarian relationship	1.17	0.86	1.02	0.98	1.13	0.89	1.03	0.97	1.07	0.94
Characteristics of proxy										
Age	1.35	0.74								
Sex	1.07	0.94								
Marital status	1.25	0.80								
Education	1.03	0.97								
Self-reported health			1.02	0.98						
Characteristics of centenarians										
Age					1.01	0.99				
Living condition					1.13	0.88				
Self-reported health					1.02	0.98				
Proxy respondents' behaviour during the interview										
Cooperation							1.03	0.97		
Ask for clarifications							1.00	0.99		
Data collection features										
Year of interview									1.16	0.86
Language-speaking region									1.10	0.90

Notes. VIF: Variance inflation factor. TC: Tolerance criterion.
