# Supplementary material for: Data quality in centenarian research: The proxy-centenarian relationship and item nonresponse in the SWISS100 study
Source: PLoS One. 2025 Jan 27;20(1):e0311847. doi: 10.1371/journal.pone.0311847 (PMC11771874; doi:10.1371/journal.pone.0311847)
Supplement: S2 Table — (PDF) [file pone.0311847.s002.pdf]

S2 Tab. Distribution of unanswered questions across variables

	n	%
Sociodemographic		
Age	0	0.0
Sex	0	0.0
Marital status	0	0.0
Education	6	8.0
Living situation	0	0.0
Self-reported health	0	0.0
Geriatric Depression Scale		
Do you feel that centenarian's life is empty?	6	8.0
Does centenarian often get bored?	4	5.3
Does centenarian often feel helpless?	7	9.3
Does centenarian feel that her/his situation is hopeless?	5	6.7
Has centenarian often felt dejected or sad?	5	6.7
Valuation of life		
Is centenarian's life guided by strong religious or other beliefs?	0	0.0
Does centenarian have a strong will to live right now?	2	2.7
Does life have meaning for centenarian?	7	9.3
Total	42	4.0
