# Supplementary material for: Data quality in centenarian research: The proxy-centenarian relationship and item nonresponse in the SWISS100 study
Source: PLoS One. 2025 Jan 27;20(1):e0311847. doi: 10.1371/journal.pone.0311847 (PMC11771874; doi:10.1371/journal.pone.0311847)
Supplement: S3 Table — (PDF) [file pone.0311847.s003.pdf]

Table S3. Firth's logistic regression models showing the association of item nonresponse with proxy-centenarian relationship, controlled for potential confounders

	M0		M1		M2		M3		M4	
	OR	95% CIs	OR	95% CIs	OR	95% CIs	OR	95% CIs	OR	95% CIs
**Proxy-centenarian relationship**										
Children [a]										
Other relatives	1.84	[0.44-7.77]	2.94	[0.55-15.70]	1.97	[0.43-8.91]	2.47	[0.54-11.30]	1.98	[0.43-9.15]
Non-relatives	6.83**	[1.96-23.81]	12.62**	[1.86-85.47]	6.63*	[1.70-25.85]	10.60**	[2.42-46.39]	6.25**	[1.67-23.35]
Control variables										
**Characteristics of proxy**										
Age			0.99	[0.92-1.06]						
Sex										
Men [a]										
Women			2.76	[0.65-11.70]						
Marital status										
Without partner [a]										
Married			3.99	[0.66-24.34]						
Widow/er			4.17	[0.24-71.03]						
Education										
Less than high school [a]										
High school, university			2.01	[0.47-8.57]						
Self-reported health										
Poor, fair [a]										
Good			0.7	[0.11-4.31]						
Very good, excellent			0.23	[0.02-2.12]						
**Characteristics of centenarians**										
Age					0.90	[0.68-1.19]				
Gender										
Men [a]										
Women					0.49	[0.11-2.15]				
Living situation										

Home [a]										
Nursing home					2.87	[0.62-13.27]				
Self-reported health										
Poor, fair [a]										
Good					0.57	[0.18-1.86]				
Very good, excellent					0.26	[0.04-1.85]				
**Proxy respondents' behaviour during the interview**										
Cooperation										
Very good [a]										
Good, fair							0.88	[0.21-3.65]		
Clarifications										
Never [a]										
At least one or twice							0.93	[0.27-3.18]		
**Data collection features**										
Interview period										
2020-2021 [a]										
2022									0.91	[0.25-3.37]
Language-speaking region										
French [a]										
German									1.39	[0.39-4.91]
Italian									0.86	[0.20-3.79]
N	75		67		75		66		75	

Notes: OR = Odds ratio. [a] = reference category.
Significance level: ** = p<.010; * = p<.050
