# Supplementary figures and images for: Data quality in centenarian research: The proxy-centenarian relationship and item nonresponse in the SWISS100 study
Source: PLoS One. 2025 Jan 27;20(1):e0311847. doi: 10.1371/journal.pone.0311847 (PMC11771874; doi:10.1371/journal.pone.0311847)

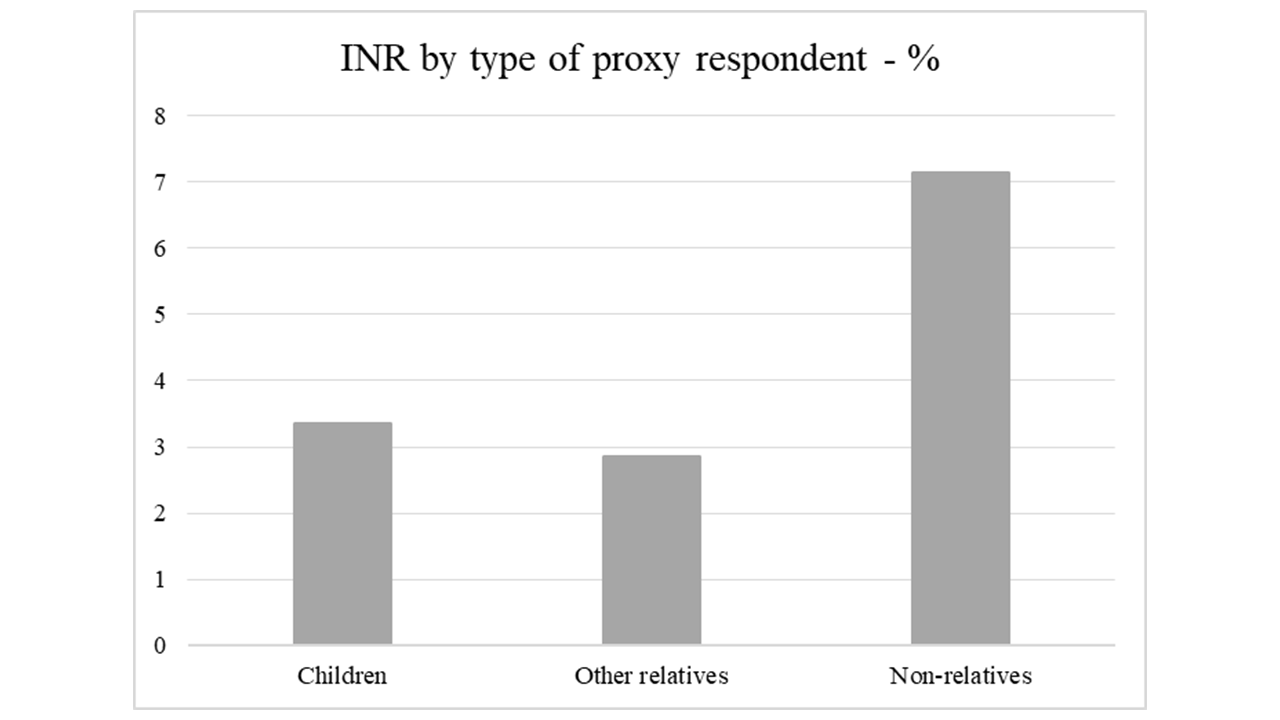

Supplement: S1 Fig — (TIF) [file pone.0311847.s004.tif]

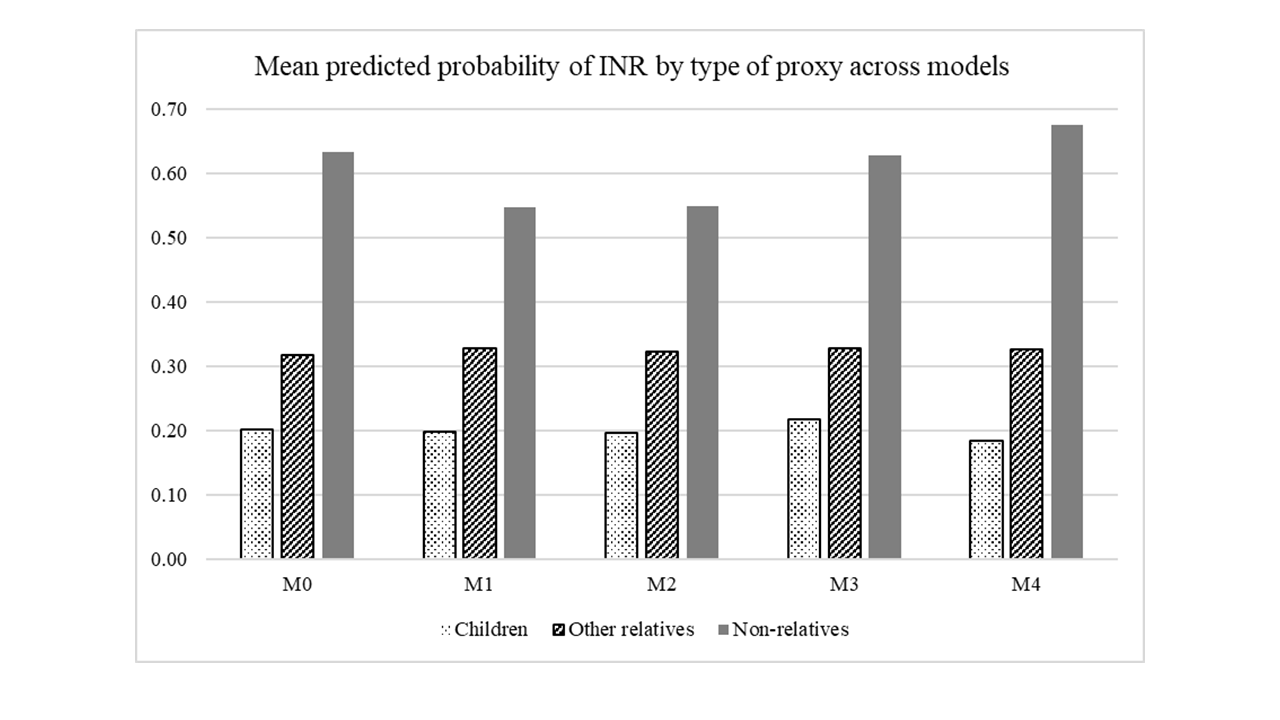

Supplement: S2 Fig — (TIF) [file pone.0311847.s005.tif]
