# Supplementary material for: Data quality in centenarian research: The proxy-centenarian relationship and item nonresponse in the SWISS100 study
Source: PLoS One. 2025 Jan 27;20(1):e0311847. doi: 10.1371/journal.pone.0311847 (PMC11771874; doi:10.1371/journal.pone.0311847)
Supplement: Attachment — Submitted filename: Reviwer comments.docx [file pone.0311847.s006.docx]

In the research paper titled "Data Quality in Centenarian Research: The Proxy-Centenarian Relationship and Item Nonresponse in the SWISS100 Study," the author explores the relationship between proxies and centenarians. The article adeptly examines mental health among the elderly.

1. The paper is professionally written, but there are some formatting issues such as inconsistent text borders and lack of justification.
2. Additionally, the referencing style varies, noticeable in the discrepancy with the 6th reference compared to others.
3. Overall, I recommend acceptance pending minor revisions.
